# Integrated Bioinformatic Analysis Identifies *TIPIN* as a Prognostic Biomarker in Hepatocellular Carcinoma

**DOI:** 10.1155/2022/5764592

**Published:** 2022-01-17

**Authors:** Hui Chen, Chunting Zhang, Qianmei Zhou, Yanan Guo, Zhigang Ren, Zujiang Yu

**Affiliations:** ^1^Precision Medicine Center, The First Affiliated Hospital of Zhengzhou University, Zhengzhou, China; ^2^Department of Infectious Diseases, The First Affiliated Hospital of Zhengzhou University, Zhengzhou, China; ^3^Department of Breast Surgery, The First Affiliated Hospital of Zhengzhou University, Zhengzhou, China; ^4^Department of Infectious Diseases, Zhengzhou Central Hospital, Zhengzhou, China

## Abstract

**Background:**

Gene expression and DNA methylation analyses have long been used to identify cancer markers. However, a combination analysis of the gene expression and DNA methylation has yet to be performed to identify potential biomarkers of hepatocellular carcinoma (HCC).

**Methods:**

By matching gene expression profiles and promoter methylation data in The Cancer Genome Atlas (TCGA), genes with discrepant expression as well as genes with differential promoter methylation were identified. High-expression genes with low promoter methylation were defined as epigenetically induced (EI), while low-expression genes with high promoter methylation were defined as epigenetically suppressed (ES). The human protein interaction network was further integrated to construct the EI/ES gene interaction network, and the key genes in the subnet were identified as potential HCC biomarkers. The expression differences and prognostic values were verified in TCGA and Gene Expression Omnibus (GEO) databases, as well as with tissue chip technology.

**Results:**

Four key genes were identified: *TIPIN*, *RBM15B*, *DUSP28*, and *TRIM31*, which demonstrated the differential gene expression and prognostic value in TCGA and GEO databases. Tissue microarray analysis (TMA) revealed that TIPIN levels were altered in HCC. The upregulated TIPIN expression was associated with worse overall survival. Univariate and multivariate analyses showed that the TIPIN expression was an independent predictor of HCC.

**Conclusion:**

*TIPIN* might be a potential novel prognostic biomarker for HCC.

## 1. Introduction

Liver cancer is the second leading cause of cancer-related deaths worldwide, with an increasing incidence in Asia and Africa [1]. Hepatocellular carcinoma (HCC) accounts for 70%–90% of liver cancers worldwide. Surgical resection provides the highest probability of long-term survival [2]; however, only 10% to 30% of patients are eligible for curative surgery because of late diagnosis [3], and the postoperative five-year survival rate is low [4]. The dismal clinical outcome of HCC is largely due to a particularly high rate of postsurgical recurrence and metastasis [5, 6]. Thus, studies aimed at identifying novel HCC biomarkers to improve the early diagnosis rate and ultimately patient survival are needed.

Hepatocarcinogenesis is a complex multistep process involving the accumulation of genetic and epigenetic alterations [7]. Epigenetic regulation by promoter methylation plays a critical role in tumorigenesis [8]. Moreover, DNA epigenetic dysregulation signatures during tumorigenesis may be used as diagnostic or prognostic markers for cancer [9]. However, the DNA methylation patterns associated with gene expression and clinical prognosis in HCC remain to be elucidated.

In this study, promoter methylation and gene expression profiles from The Cancer Genome Atlas (TCGA) were used to screen candidate genes associated with HCC prognosis. We identified four genes: RNA binding motif protein 15B (*RBM15B*), timeless- (TIM-) interacting protein (*TIPIN*), dual-specificity phosphatase 28 (*DUSP28*), and tripartite motif 31 (*TRIM31*). Subsequently, the expression difference was verified using Gene Expression Omnibus (GEO), and survival analysis was used to identify prognosis biomarkers of liver cancer. The relationship between these genes and HCC stages was validated using TCGA data, and subsequently, we focused on the gene *TIPIN*. Furthermore, the relationship between the gene expression of *TIPIN* and clinical features was demonstrated with a tissue microarray (TMA) of a cohort of patients with HCC (*n* = 160).

## 2. Materials and Methods

### 2.1. DNA Methylation and Gene Expression Profile Downloading and Processing

High-throughput data for RNA sequencing (RNA-seq) and DNA methylation and clinical characteristics of the liver cancer cohort were downloaded from TCGA (https://tcga-data.nci.nih.gov/tcga/). By matching methylation data and RNA-Seq data, 41 pairs of samples contained cancer, and paracancerous samples were obtained. Methylation data were measured on the 450 K array and quantified using beta values. The methylation probe was mapped to the promoter region of the gene, which was defined as 800 bp upstream to 200 bp downstream of *TSS*, and the empty probe was removed. When multiple probes corresponded to one gene, we used the average as the gene promoter methylation level. The RNA-seq data were measured by transcripts per million (TPM) transformed from fragments per kilobase million (FPKM) before the subsequent analysis.

### 2.2. Identification and Integration Analysis of Differential DNA Methylation and Gene Expression in HCC

The average methylation values at the CpG site and gene expression values were compared between the liver cancer and normal groups using Wilcoxon's test for nonpaired samples. The following criteria were used to calculate the differential expression: log2 (fold change) > 1 and FDR-corrected *P* value <0.05. To calculate the differential promoter methylation, we used *P* value <0.05.

We defined genes with the high expression and low promoter methylation as epigenetically induced (EI) and genes with low expression and high promoter methylation as epigenetically suppressed (ES). We then analyzed the relationship between differentially expressed genes and genes with differential promoter methylation using a Venn diagram and computed Spearman correlation coefficients between promoter methylation level and expression for the candidate genes. Gene enrichment tests were performed on the candidate genes. ClusterProfiler (version 3.4.4) was used to detect significant enrichment for Gene Ontology (GO) and Kyoto Encyclopedia of Genes and Genomes (KEGG) pathways. All correlation analyses were performed using the Limma *R* package 2.12.0.

### 2.3. Interaction Control Network Construction and Analysis of Network Degree Distribution

Human protein interaction data were downloaded from the HIPPIE database (https://www.ncbi.nlm.nih.gov/pmc/articles/PMC5210659) to construct a human protein interaction network. The obtained EI and ES genes were mapped into a human protein interaction network to construct an EI/ES gene interaction subnetwork.

We set the human protein interaction network as the statistical background and counted the number of genes and EI/ES genes interacting with each EI and ES genes. We then constructed a statistical model using Fisher enrichment testing for each EI/ES gene. The following criteria were used to select the key epigenetically altered driver genes: (1) FDR < 0.05 and (2) the proportion of EI/ES genes in the neighbor node of the gene was more than 10%.

### 2.4. GEO Dataset Processing and Analysis

Twenty sets of microarrays were obtained from the GEO database (http; //http://www.ncbi.nlm.nih.gov/geo/) and were used to demonstrate target gene expression. The 20 cohorts were compounded of GSE6764, GSE14520, GSE36376, GSE39791, GSE45436, GSE54236, GSE54238, GSE57957, GSE60502, GSE62232, GSE64041, GSE76297, GSE76427, GSE25097, GSE77314, GSE84005, GSE84598, GSE102083, GSE10143, and GSE14811. The characteristics including cohort ID, RNA-seq platform, number of samples (cancer and noncancer samples), publication year, and country are summarized in Table S1.

### 2.5. Patients and Specimens

TMAs containing 80 pairs of HCC specimens and corresponding nontumor tissues obtained from the First Affiliated Hospital of Zhengzhou University in 2015 to 2019 were constructed using a 1.5 mm diameter core. All specimens were collected from patients who underwent surgical resection at the Hospital, and all pathological data were retrieved from the pathology department. Diagnosis was confirmed by two pathologists, according to the American Joint Committee on Cancer TNM staging classification for carcinoma of the liver (7^th^ ed., 2011). We have obtained the approval of the Institutional Review Board of the First Affiliated Hospital of Zhengzhou University. Written informed consent was obtained from each patient, and this study complied with the Declaration of Helsinki.

### 2.6. Immunohistochemical Staining

TMA sections, with 5 *μ*m thickness, were dewaxed, treated with hydrogen peroxide to quench endogenous peroxidase activity, and then incubated with rabbit anti-human antibody (1 : 100; Proteintech, Wuhan, China) at 4°C overnight. Biotinylated goat anti-rabbit secondary antibodies (1 : 200; Proteintech, Wuhan, China) were then added. Finally, the samples were treated with DAB solution for 3 min to visualize the staining. Cells containing brown granules were independently counted by two pathologists who were blinded to the clinical parameters. According to the staining intensity, the samples were scored as 1, weak; 2, moderate; 3, strong; and 4, intense. Scores of 1–2 and 3–4 were defined as low expression and high expression, respectively.

### 2.7. Statistical Analysis

Statistical analysis was performed using SPSS software (version 25.0) or *R* (version 3.6.1). The Wilcoxon matched-pair signed-rank test was used to compare protein expression differences between carcinoma and paracancerous tissues of HCC patients. The chi-squared test was used to determine the association between gene expression levels and clinicopathological characteristics. The Kaplan–Meier method was used for survival analysis. Univariate and multivariate Cox proportional hazard regression models were used to analyze prognostic factors. Pearson's correlation coefficient was used to assess the linear association between the two variables.

## 3. Results

### 3.1. Integrated Analysis of DNA Methylation and Gene Expression Profiles

**Figure 1:**
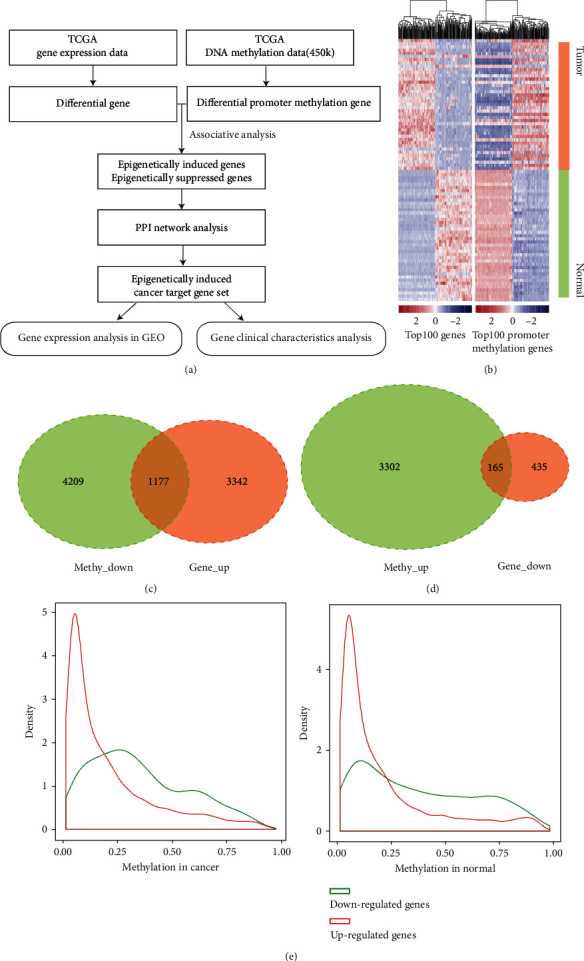
Integrated analysis of DNA methylation and gene expression profiles in TCGA. Notes: (a) The flowchart of the analysis process on selection of the epigenetically altered driver genes. (b) The cluster map of the mRNA expression of the top 100 genes and top 100 genes with promoter methylation in TCGA cohort. (c, d) Venn diagram of differentially expressed genes and genes with differential promoter methylation. (e) The negative relationship between gene expression and promoter methylation levels in cancer and normal tissues.

As shown in the analysis flow chart in Figure 1(a), we obtained RNA-seq and methylation data for 41 pairs of cancer and paracancerous samples from TCGA datasets. There are 17,937 genes that showed promoter methylation. From this gene set, we obtained 5,119 genes that showed the discrepant gene expression in cancer and adjacent cancer samples, according to the principle of differential screening. Of these, 4,519 genes were upregulated, and 600 genes were downregulated. Moreover, promoter methylation of 8,853 genes was differentially expressed in cancer and paracancer samples; of these genes, 3,467 promoter methylation were upregulated, and 5,386 were downregulated. The top 100 most distinguishing genes are shown in Figure 1(b). It is well known that DNA promoter methylation and gene expression have a negative correlation, as high methylation level inhibits the expression of the downstream gene, and low methylation level promotes the gene expression [10]. Therefore, we defined two concepts: low promoter methylation and high expression gene were epigenetically induced gene (EI); high promoter methylation and low expression gene were epigenetically suppressed gene (ES). The Venn diagram in Figures 1(c) and 1(d) shows 1,177 EI and 165 ES genes by combining genes with differential promoter methylation and differentially expressed genes.  Figure 1(e) shows that the higher the promoter methylation level in cancer and normal tissues, the lower the expression of differentially expressed genes, indicating that promoter DNA methylation was negatively correlated with the gene expression.

### 3.2. Selection of the Epigenetically Altered Driver Genes

**Figure 2:**
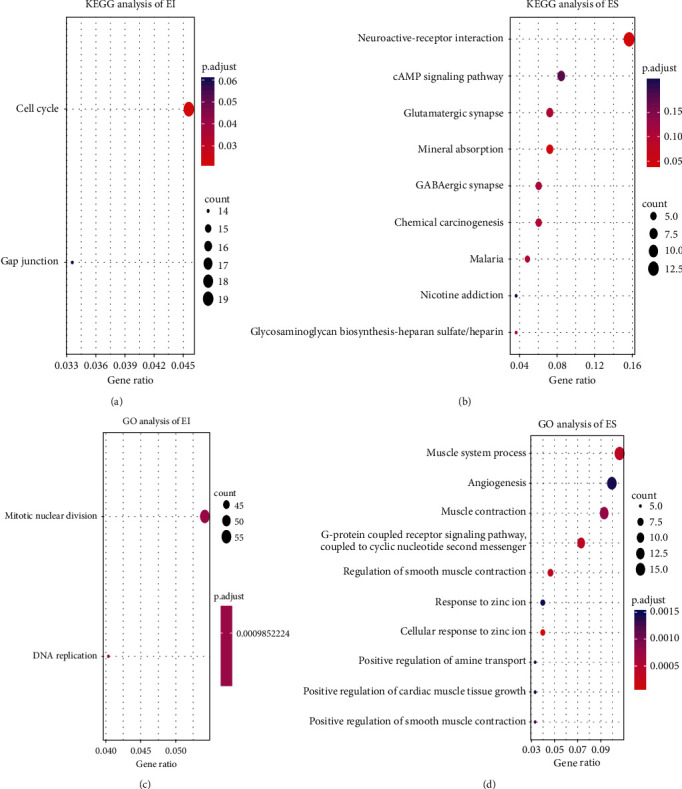
The consequence of KEGG and GO enrichment analysis of EI/ES genes in TCGA. Notes: (a) KEGG enrichment analysis of EI genes. (b) KEGG enrichment analysis of ES genes. (c) GO enrichment analysis of EI genes. (d) GO enrichment analysis of ES genes.

**Figure 3:**
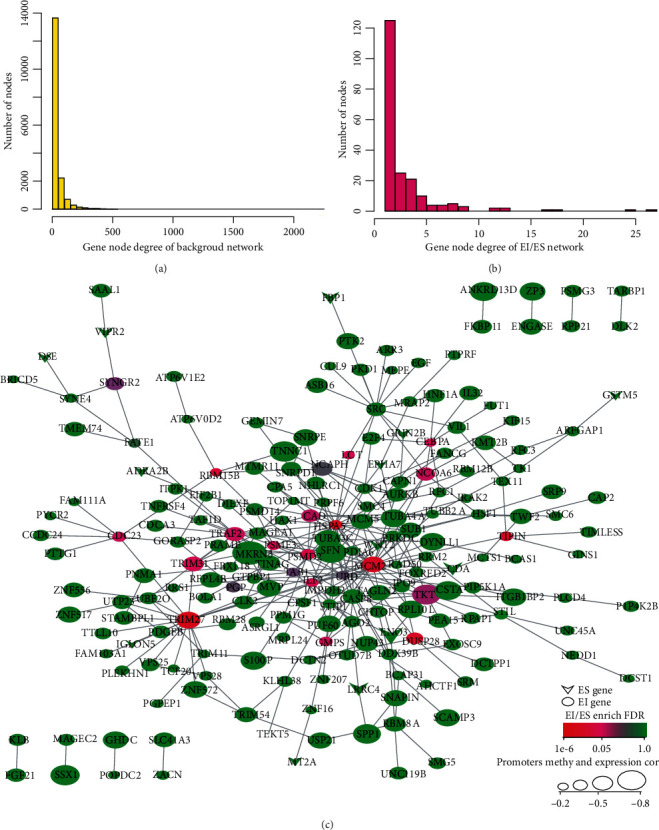
Degree distribution of network and EI/ES gene interaction network. Notes: (a) Degree distribution of background network. (b) Degree distribution of EI/ES gene interaction network. (c) EI/ES gene interaction network.

KEGG and GO enrichment analyses were performed to elucidate the biological functions of 1,177 EI and 165 ES genes (Figure 2). We observed that EI genes mainly gather in the cell cycle, gap junction, DNA replication, and mitotic nuclei, which have been reported to be associated with the occurrence of cancer (Figures 2(a) and 2(c)). Conversely, ES genes mainly gather in mineral absorption, glutamatergic synapse, GABAergic synapse, and cAMP signaling pathway, which are necessary for normal cell function and integrity (Figures 2(b) and 2(d)). By calculating the correlation of EI or ES genes between gene expression and promoter methylation, we obtained 419 EI and 68 ES genes with a negative correlation. Moreover, we loaded human protein interaction data from the HIPPIE database to construct protein interactive networks, including 17,381 nodes and an average of 19.6 neighbor nodes. Furthermore, EI and ES genes were mapped to the human protein internetworks to structure EI/ES gene interaction networks, where 436 genes were mapped to the network and included 315 nodes, with an average of 1.54 neighbor nodes.  Figure 3(c) shows that the number of ES genes are few than EI genes, and the enrichment degree of the EI/ES genes is mostly low (the green circles are more). Finally, we chose *TIPIN*, *RBM15B*, *DUSP28*, and *TRIM31* (Table 1) as epigenetically altered driver genes, in which the proportion of EI/ES genes in the neighbor node of the gene was more than 10%, and the FDR of *P* value in the enrichment significance of EI/ES genes was less than 0.05. As shown in Figure S1A, a high correlation was found between promoter methylation level and expression of the expression-specific genes. Additionally, *DUSP28* and *TRIM31* have been reported to be associated with liver cancer.

### 3.3. The Prognostic Value of the Epigenetically Altered Driver Genes Determined Using TCGA and GEO

**Figure 4:**
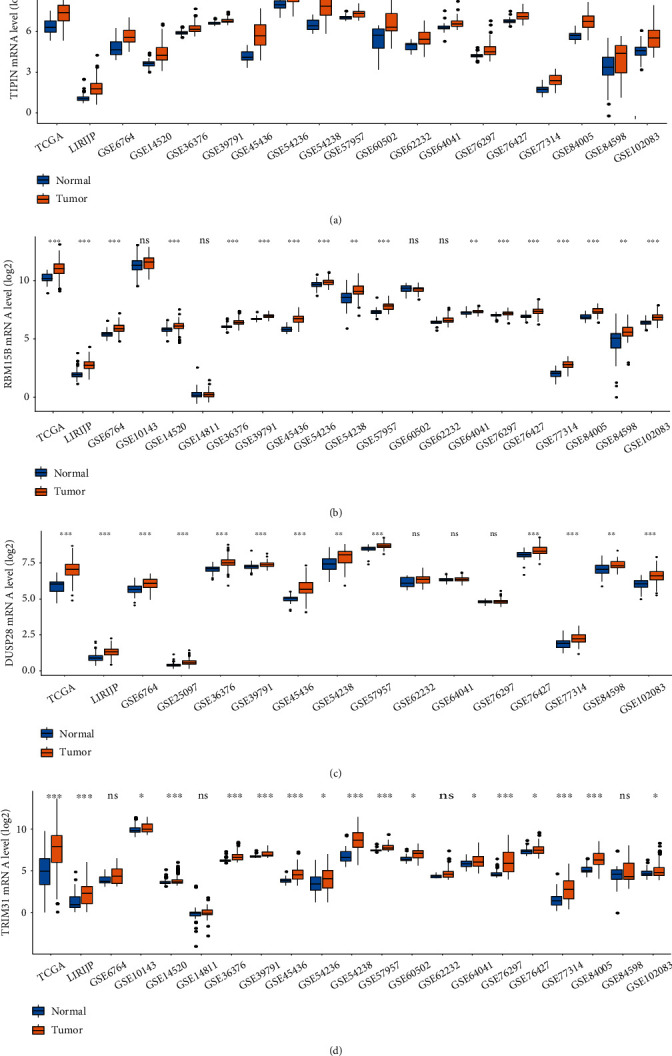
Expression of four expression-specific mRNAs in databases. Notes: The mRNA expression of (a) *TIPIN*, (b) *RBM15B*, (c) *DUSP28*, and (d) *TRIM31* in databases. ^∗^*P* < 0.05, ^∗∗^*P* < 0.01, ^∗∗∗^*P* < 0.001, ^ns^*P* > 0.05.

**Figure 5:**
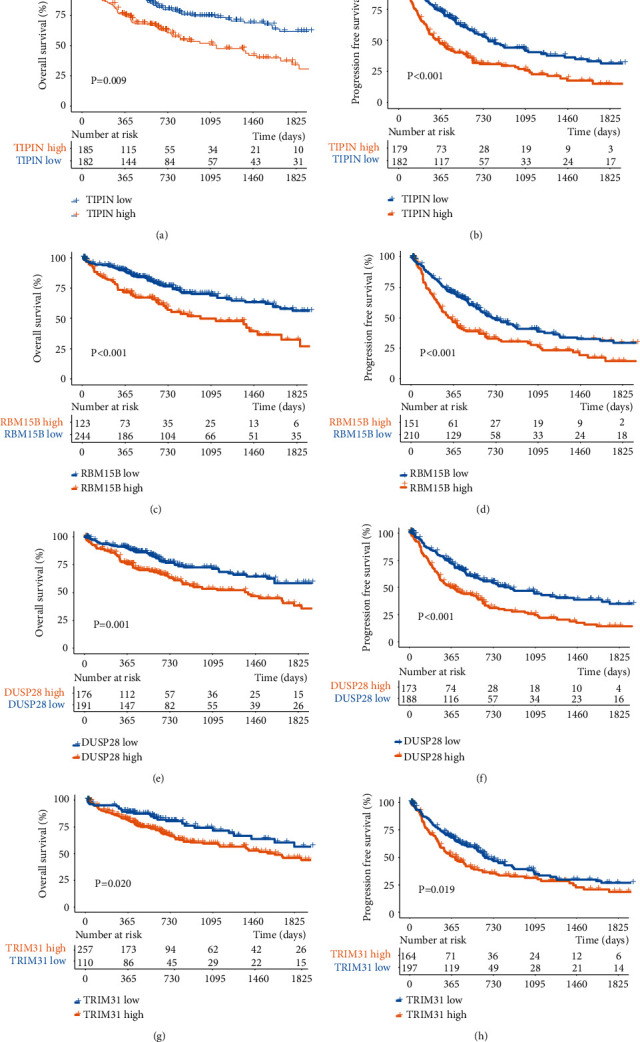
Survival analysis based on mRNA expression of four expression-specific genes. Notes: (a) OS analysis with the *TIPIN* expression. (b) PFS analysis with the *TIPIN* expression. (c) OS analysis with the *RBM15B* expression. (d) PFS analysis with the *RBM15B* expression. (e) OS analysis with the *DUSP28* expression. (f) PFS analysis with the *DUSP28* expression. (g) OS analysis with the *TRIM31* expression. (h) PFS analysis with the *TRIM31* expression.

To explore the prognostic value of the four genes, data were retrieved from TCGA and GEO. The mRNA expression data were used to determine differential gene expression (Figure 4). The results show that the mRNA expression of the four genes is significantly upregulated in cancer samples compared to normal samples. Additionally, we analyzed the correlation between gene expression and the survival rate (Figure 5). The results suggest that all four expression-specific genes might be prognostic biomarkers of liver cancer.

**Figure 6:**
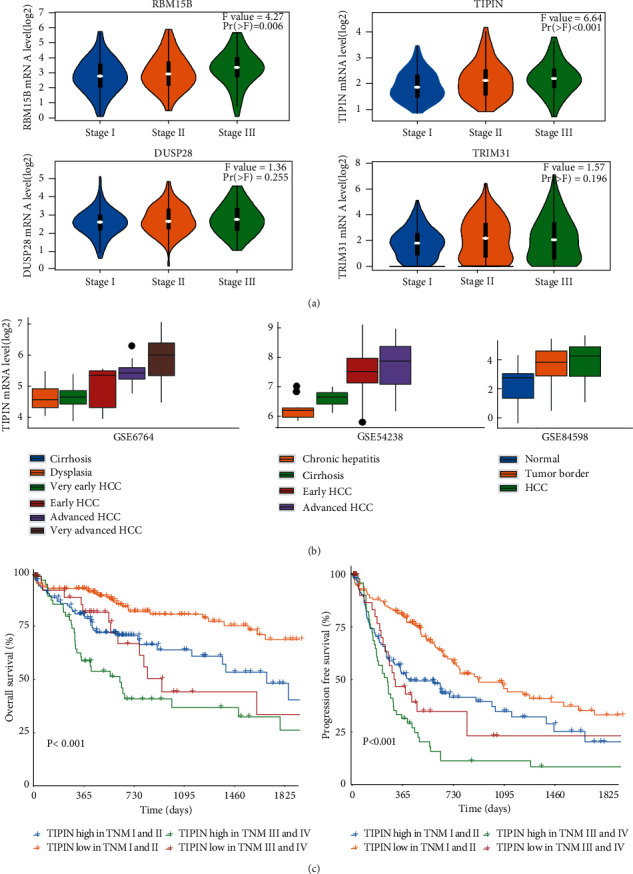
The expression of four expression-specific mRNAs during the development of HCC. Notes: (a) Expression of four specific mRNAs during different stages of liver cancer in TCGA. (b) The *TIPIN* expression during the development of liver disease in GEO databases. (c) Overall survival analysis of patients with different TNM stages, based on the *TIPIN* expression in TCGA.

To observe the changes in expression of the four genes during the development of cancer, we used TCGA data to analyze their expression at different stages (Figure 6(a)). *RBM15B* and *TIPIN* showed significant differences in the stages of cancer development. Moreover, during the progression of liver disease, the *TIPIN* mRNA expression was subsequently increased, as shown in Figure 6(b). This suggests that *TIPIN* may promote the progression of liver disease. Meanwhile, patients with an advanced TNM stage and high *TIPIN* expression had a poor prognosis (Figure 6(c)). Overall, our results showed that the four specific genes, *TIPIN*, *RBM15B*, *DUSP28*, and *TRIM31*, were likely prognostic biomarkers of liver cancer, and *TIPIN* might conspicuously accelerate the process of hepatocarcinogenesis. In addition, the results of ROC analysis revealed a significant diagnostic value of *TIPIN* in HCC (Figure S2).

### 3.4. Upregulated TIPIN Is Associated with Clinicopathological Characteristics

**Figure 7:**
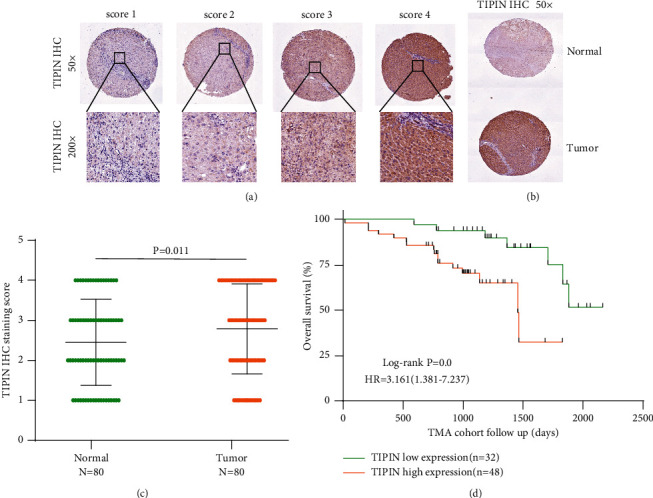
High TIPIN levels were negatively correlated with survival in HCC. Notes: (a) Representative images of TIPIN IHC staining in HCC tissues. (b) Representative TIPIN staining in HCC and adjacent tissues. (c) TIPIN levels are increased in HCC tissues compared to adjacent tissues. (d) Kaplan-Meier analysis shows that upregulated TIPIN levels are correlated with poor prognosis in patients with HCC.

To further verify the prognostic value of TIPIN for HCC, 80 pairs of cancer and paracancerous samples and associated clinical information were collected. Tissue samples were used for immunohistochemical staining, and clinical data were used for correlation analysis and survival analysis. According to the staining intensity, TIPIN staining was scored from 1 to 4 (Figure 7(a)). A score of 1 to 2 was defined as the low *TIPIN* expression, whereas a score of 3 to 4 was defined as the high *TIPIN* expression. The results showed that the TIPIN level was altered between cancer and paracancerous tissues (Figures 7(b) and 7(c)). High TIPIN levels were associated with poor prognosis, based on the overall survival (OS) analysis (*P* = 0.011, Figure 7(d)). We then analyzed the relationship between TIPIN levels and clinical characteristics, and the results indicated that TIPIN levels were correlated with TNM stages (Table 2). Univariate and multivariate survival analyses showed that TIPIN levels and TNM stage were independent prognostic factors for HCC (Table 3 and Table S2). In addition, we conducted gene set enrichment analysis (GSEA) based on the Molecular Signatures Database (MSigDB) to preliminarily explore the molecular mechanism of *TIPIN* in HCC progression (Figure S3).

## 4. Discussion

DNA methylation drives epigenetic regulation by methyltransferase-mediated catalysis from CG nucleotide cytosine to 5-methylcytosine [11]. Dysfunction of DNA methylation can lead to activation of oncogenes and inactivation of tumor suppressor genes; abnormal DNA methylation is often acted as a characteristic of malignant tumors [12, 13]. In comparison to genetic mutations, DNA methylation is a more potential therapeutic target in malignancies, because of the reversibility of epigenetic modifications [14]. Currently, many drugs like 5-azacytidine (5-AZA) and decitabine are in clinical trials in HCC, which may alter DNA methylation patterns or levels [15]. Previous studies have shown that abnormal DNA methylation is closely related to the occurrence, diagnosis, prognosis, and treatment of liver cancer [16, 17]. For example, DNA methylation interferes with the expression of the deleted in the liver cancer 1 (*DLC-1*) gene, leading to the initiation of HCC as the gene encodes a tumor suppressor [18]. Other genes encoding tumor suppressors could also be silenced by DNA methylation, such as Ras association domain family 1A (*RASSF1A*) [19], human runt-related transcription factor 3 (*RUNX3*) [15], multiple tumor suppressor 1 (*p16*) [20], and suppressor of cytokine signaling 1 (*SOCS-1*) [21, 22]. The expression of the gene encoding Bcl-2-like protein 10 (*BCLB*) could be reduced by DNA methylation, and the decreased *BCLB* expression might be a therapeutic target for HCC because the gene is involved in HCC development [23]. It is reported that after sorafenib treatment—the only treatment option for unresectable and advanced HCC, oncogenes were prone to hypermethylation, and the tumor suppressor genes were apt to be hypomethylated in human-derived hepatoma cell line (HA22T/VGH), such as the hypermethylation of Janus kinase (JAK1) gene and the hypomethylation of SMAD family member 2 (SMAD2) gene [24]. Abovementioned all indicate that abnormal DNA methylation plays a coordinating role in promoting the initiation and development of HCC. Moreover, DNA methylation biomarkers may become targets for the diagnosis and treatment of HCC.

There are few studies on the combination of DNA methylation analysis with gene expression to search for tumor molecular markers. In our study, integrated analysis of gene expression and DNA methylation identified four genes: *TIPIN*, *RBM15B*, *DUSP28*, and *TRIM31*. As a member of the atypical *DUSP* family, *DUSP28* has been reported to be significantly upregulated in HCC tissues and cell lines and plays an important role in HCC progression [25]. *TRIM31* belongs to the tripartite motif-containing or RING, B-box, and coiled-coil family; it acts as a tumor promoter and has been shown to promote HCC progression [26]. However, the relationship between *TIPIN* or *RBM15B* and HCC has not been reported. In our study, the data obtained from TCGA and GEO datasets showed the differential expression and prognostic value of the four epigenetically altered driver genes. Simultaneously, it was demonstrated that the RNA expression of *TIPIN* and *RBM15B* was discrepant during the development of HCC. Moreover, TMA analysis revealed that TIPIN levels varied between cancerous and paracancerous tissues. Based with clinical information, the prognosis of the patients with high the TIPIN expression was worse than that of those with the low TIPIN expression. *TIPIN* is associated with TNM stage and could be used as an independent prognostic factor for HCC.

*TIPIN* interacts with the core circadian protein TIM to form TIM-TIPIN complex, participating in normal DNA replication to maintain genomic stability [27, 28]. During the process of DNA replication, the TIM-TIPIN complex moves along the replication fork through its checkpoint adjustment function or independent adjustment function to maintain the integrity and stability of the replication fork, facilitating the DNA replication process to return to normal [29, 30]. The downregulation of the TIM-TIPIN complex results in a reduced rate of DNA synthesis [31]. It was previously reported that the *TIPIN* mRNA level is significantly upregulated in breast cancer, particularly in the most proliferative and poor prognosis-related breast cancer subtypes (triple negative breast cancer, HER2, and Luminal B). Silencing of the *TIPIN* expression induced apoptosis and inhibited proliferation in breast cancer cell lines, making *TIPIN* a potential target for breast cancer therapy [32]. Furthermore, knockdown of the TIM-TIPIN complex has been reported to promote apoptosis in melanoma cell lines [33]. However, because of the limited conditions, we could not conduct molecular, cellular, and animal experiments; therefore, the specific molecular mechanism of *TIPIN* in the progression of HCC remains to be further explored.

In conclusion, our study is the first to show that *TIPIN* is overexpressed in HCC at the mRNA as well as protein level. The high expression of *TIPIN* indicates a poor prognosis of HCC patients. *TIPIN* may be a potential prognosis signature for HCC.

## Figures and Tables

**Table 1:** The selected epigenetically altered driver.

Gene symbol	Number of EI neighbor gene	Number of neighbor gene	Number of EI gene	Number of network gene	EI neighbor gene per	Fisher's exact test *P* value	FDR
TIPIN	4	12	205	17381	0.250	3.06*E*-05	0.006
RBM15B	5	31	205	17381	0.139	6.09*E*-05	0.012
DUSP28	5	26	205	17381	0.161	2.88*E*-05	0.006
TRIM31	4	20	205	17381	0.167	1.66*E*-04	0.033

TIPIN: TIM-interacting protein; RBM15B: RNA binding motif protein 15B; DUSP28: dual specificity phosphatase 28; TRIM31: tripartite motif 31; EI: epigenetically induced genes; FDR: false discovery rate.

**Table 2:** The relationship between TIPIN expression and the clinicopathological features of HCC in TMA.

Clinicopathological features		No. of cases (%)	TIPIN expression level		*P* value
			Low	High	
Age (years)	≤55	40	20	20	0.068
	>55	40	12	28	
Gender	Female	17	10	7	0.074
	Male	63	22	41	
Pathogenesis	HBV	58	21	37	0.261
	Other	22	11	11	
ALT (U/L)	≤35	48	20	28	0.709
	>35	32	12	20	
AST (U/L)	≤31	42	15	27	0.411
	>31	38	17	21	
TB (U/L)	≤12	43	18	25	0.714
	>12	37	14	13	
ALB (G/L)	≤40	47	18	29	0.711
	>40	33	14	19	
TNM	I and II	59	30	29	0.002^∗∗^
	III and IV	21	2	19	
Tumor size	≤5 cm	40	18	22	0.361
	>5 cm	40	14	26	

TIPIN: TIM-interacting protein; ALT: alanine amiotransferase; AST: aspartate transaminase; TB: total bilirubin; ALB: albumin; TNM: tumor node metastasis; ^∗∗^*P* < 0.01.

**Table 3:** Univariate and multivariate analysis of the overall survival of HCC in TMA.

Clinicopathological features		Univariate analysis			Multivariate analysis		
		HR	95% CI	*P* value	HR	95% CI	*P* value
Age (years)	≤55	1.000	1.004-1.107	0.032^∗^	1.000	0.979-1.081	0.260
	>55	1.055			1.029		
Gender	Female	1.000	0.839-15.473	0.086			
	Male	3.604					
Pathogenesis	HBV	1.000	0.564-3.151	0.512			
	Other	1.333					
ALT (U/L)	≤35	1.000	0.259-1.535	0.310			
	>35	0.631					
AST (U/L)	≤31	1.000	0.185-1.083	0.075			
	>31	0.448					
TB (U/L)	≤12	1.000	0.685-3.607	0.286			
	>12	1.572					
ALB (G/L)	≤40	1.000	0.362-2.079	0.749			
	>40	0.867					
TNM	I and II	1.000 4.536	1.856-11.086	0.001^∗∗^	1.000	1.137-7.861	0.026^∗^
	III and IV				2.989		
Tumor size	≤5 cm	1.000	0.770-4.097	0.178			
	>5 cm	1.776					
TIPIN expression	Low	1.000	1.531-13.018	0.006^∗∗^	1.000	1.079-10.996	0.037^∗^
	High	4.465			3.444		

TIPIN: TIM-interacting protein; ALT: alanine amiotransferase; AST: aspartate transaminase; TB: total bilirubin; ALB: albumin; TNM: tumor node metastasis; HR: hazard ratio; CI: confidence interval; ^∗^*P* < 0.05; ^∗∗^*P* < 0.01.

## Data Availability

The datasets presented in this study can be found in online repositories. The names of the repository/repositories and accession numbers can be found in the article (TCGA, https://tcga-data.nci.nih.gov/tcga/;GEO,http://www.ncbi.nlm.nih.gov/geo/; accession numbers: GSE6764, GSE14520, GSE36376, GSE45436, GSE39791, GSE54236, GSE54238, GSE57957, GSE60502, GSE62232, GSE64041, GSE76297, GSE76427, GSE25097, GSE77314, GSE84005, GSE84598, GSE102083, GSE10143, and GSE14811).

## Supplementary Materials

Supplementary Materials Supplementary Table 1 the characteristics of GEO datasets. Supplementary Table 2: univariate and multivariate analysis of the overall survival of HCC in TCGA. Supplementary Figure 1: the negative relationship between promoter methylation level and gene expression level of the expression specific genes. Supplementary Figure 2: ROC curves for evaluating the diagnostic power of TIPIN in HCC. Supplementary Figure 3: molecular mechanism of TIPIN action in HCC. A GSEA of TIPIN based on the Molecular Signatures Database.
