# Supplementary material for: Integrated Bioinformatic Analysis Identifies TIPIN as a Prognostic Biomarker in Hepatocellular Carcinoma
Source: Dis Markers. 2022 Jan 17;2022:5764592. doi: 10.1155/2022/5764592 (PMC8786536; doi:10.1155/2022/5764592)
Supplement: Supplementary Materials — Supplementary Table 1 the characteristics of GEO datasets. Supplementary Table 2: univariate and multivariate analysis of the overall survival of HCC in TCGA. Supplementary Figure 1: the negative relationship between promoter methylation level and gene expression level of the expression specific genes. Supplementary Figure 2: ROC curves for evaluating the diagnostic power of TIPIN in HCC. Supplementary Figure 3: molecular mechanism of TIPIN action in HCC. A GSEA of TIPIN based on the Molecular Signatures Database. [file 5764592.f1.docx]

**Supplementary Table 1.** The characteristics of GEO datasets.

| Cohort ID | Public year | Country | RNAseq platfrom | Total | Normal | Tumor | Other |
| --- | --- | --- | --- | --- | --- | --- | --- |
| GSE6764 | 2007 | USA | Affymetrix | 75 | 35 | 35 | 0 |
| GSE14520 | 2010 | USA | Affymetrix | 488 | 255 | 220 | 13 |
| GSE36376 | 2012 | South Korea | Illumina | 433 | 193 | 240 | 0 |
| GSE45436 | 2014 | Taiwan, China | Affymetrix | 134 | 39 | 95 | 0 |
| GSE39791 | 2014 | USA | Illumina | 144 | 72 | 72 | 0 |
| GSE54236 | 2014 | Italy | Agilent | 161 | 80 | 81 | 0 |
| GSE54238 | 2014 | USA | Arraystar | 56 | 30 | 26 | 0 |
| GSE57957 | 2014 | Singapore | Illumina | 78 | 39 | 39 | 0 |
| GSE60502 | 2015 | Taiwan, China | Affymetrix | 36 | 18 | 18 | 0 |
| GSE62232 | 2014 | France | Affymetrix | 91 | 10 | 81 | 0 |
| GSE64041 | 2016 | Switzerland | Affymetrix | 125 | 5 | 120 | 0 |
| GSE76297 | 2017 | USA | Affymetrix | 304 | 151 | 153 | 0 |
| GSE76427 | 2017 | Singapore | Illumina | 167 | 52 | 115 | 0 |
| GSE25097 | 2011 | USA | Rosetta/Merck | 557 | 243 | 268 | 46 |
| GSE77314 | 2016 | China | Illumina | 100 | 50 | 50 | 0 |
| GSE84005 | 2017 | China | Affymetrix | 76 | 38 | 38 | 0 |
| GSE84598 | 2017 | Germany | Illumina | 66 | 22 | 22 | 22 |
| GSE102083 | 2018 | Japan | Affymetrix | 257 | 91 | 152 | 14 |
| GSE10143 | 2008 | USA | DASL | 387 | 307 | 80 | 0 |
| GSE14811 | 2009 | South Korea | KRIBB | 112 | 56 | 56 | 0 |

**Supplementary Table 2.** Univariate and multivariate analysis of the overall survival of HCC in TCGA.

| Clinicopathological  features | | | Univariate analysis | | | | Multivariate analysis | | | | | |
| --- | --- | --- | --- | --- | --- | --- | --- | --- | --- | --- | --- | --- |
|  |  |  | HR 95%CI P value | | | | HR 95%CI P value | | | | | |
| Age(years) | | ≤median  >median | 1.000  1.218 | 0.856-1.733 | 0.274 | |  | |  | | |  |
| Gender | Female  male | | 1.000  0.812 | 0.567-1.164 | | 0.257 |  | | | | | |
| Rice | white | | 1.000  1.202 | 0.835-1.732 | | 0.322 |  |  | | |  | |
|  | other | |  |  |  |  |  |  |  |  |  |  |
| TNM | I and II  III and IV | | 1.000  2.506 | 1.721-3.650 | | <0.001*** | 1.000  2.418 | | | 1.648-3.547 | <0.001*** | |
| TIPIN expression | ≤median  >median | | 1.000  1.610 | 1.126-2.302 | | 0.009** | 1.000  1.606 | 1.090-2.369 | | | 0.017* | |

TIPIN: TIM-interacting protein; TNM: tumour-node-metastasis; HR: hazard ratio; CI: [confidenceinterval](javascript:;); *P<0.05; **P<0.01; ***P<0.001.


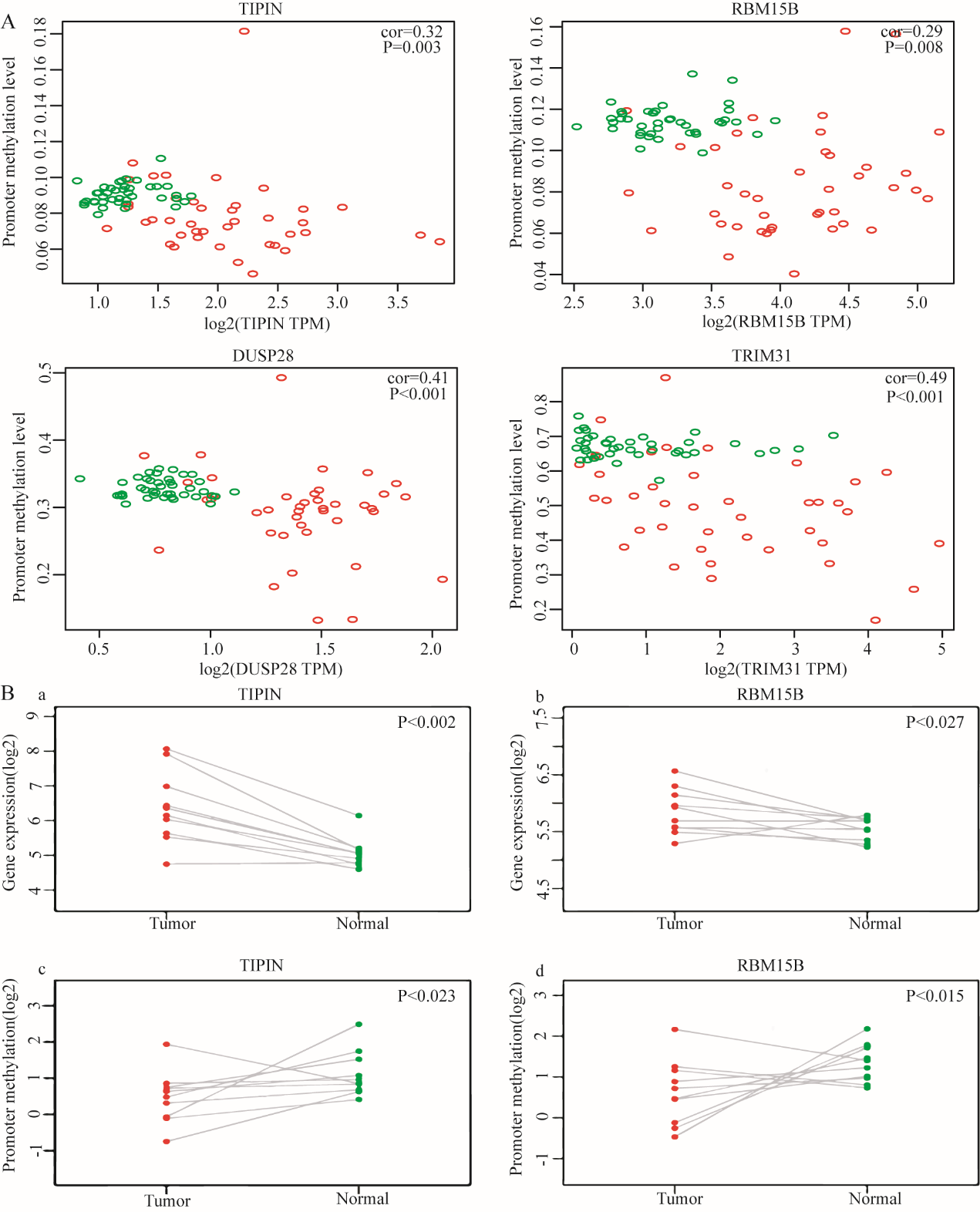


**Supplementary Figure 1.** **The negative relationship between promoter methylation level and gene expression level of the expression specific genes**

**Notes:** **(A)**The negative relationship between promoter methylation and gene expression in TCGA. Red dot represents cancer sample and green dot represents para-carcinoma sample. **(B)** a,b：gene expression significance of *TIPIN* and *RBM15B* in GSE29722 dataset; c,d: promoter methylation difference of *TIPIN* and *RBM15B* in GSE29722 dataset.


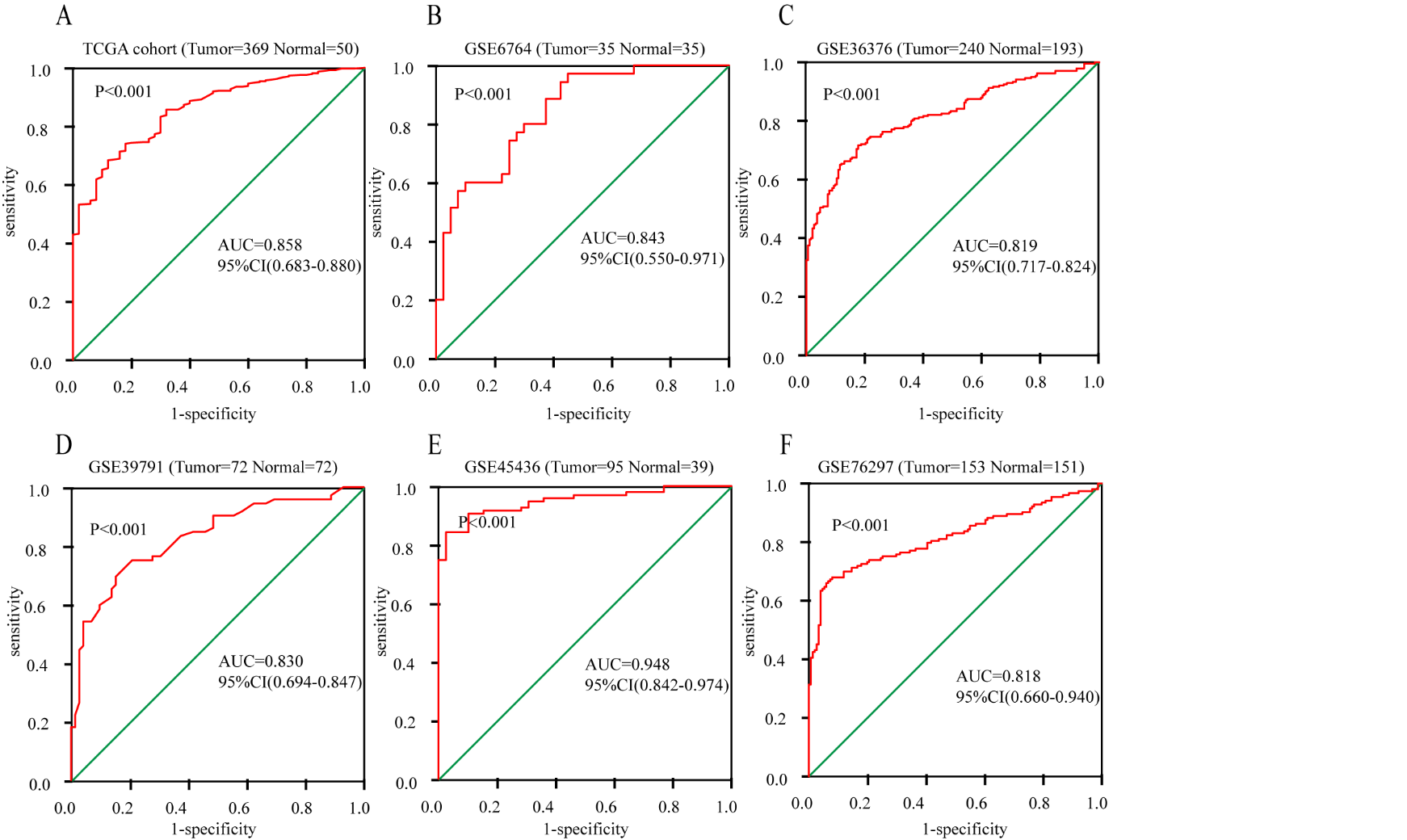


**Supplementary Figure 2. ROC curves for evaluating the diagnostic power of TIPIN in HCC**

**Notes:** **(A)** ROC assay shows the high diagnostic value of TIPIN in TCGA cohort. **(B)** ROC assay in GSE6764. **(C)** ROC assay in GSE36376. **(D)** ROC assay in GSE39791. **(E)** ROC assay in GSE45436. **(F)** ROC assay in GSE76297.


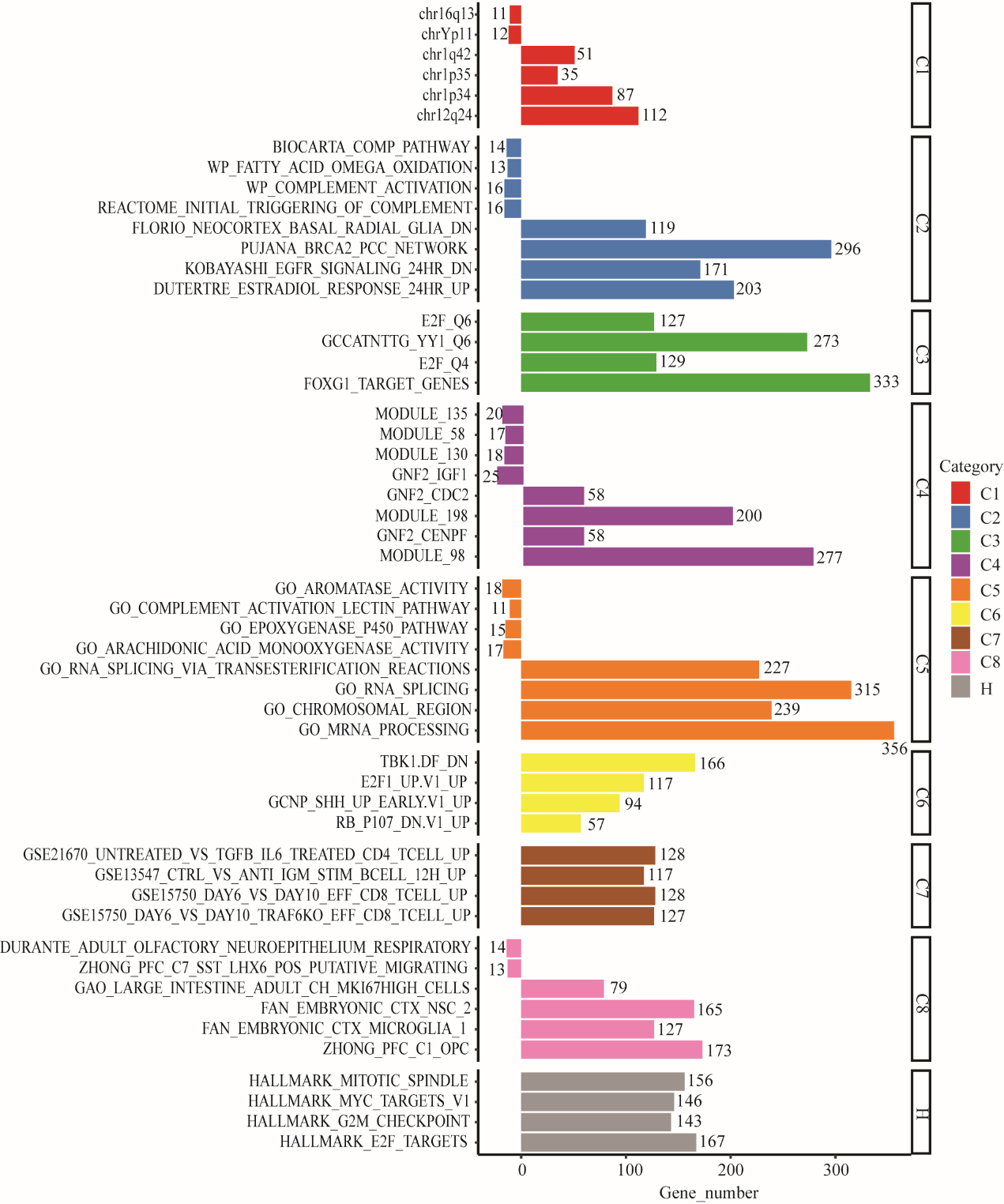


**Supplementary Figure 3.** **Molecular mechanism of TIPIN action in HCC.**

**Notes:** A GSEA of TIPIN based on the Molecular Signatures Database.
